# Plant-derived natural products targeting ion channels for pain

**DOI:** 10.1016/j.ynpai.2023.100128

**Published:** 2023-04-17

**Authors:** Sachin Goyal, Shivali Goyal, Aleyah E. Goins, Sascha R.A. Alles

**Affiliations:** a Department of Anesthesiology and Critical Care Medicine, University of New Mexico School of Medicine, Albuquerque, NM 87106, USA; b School of Pharmacy, Abhilashi University, Chail Chowk, Mandi, HP 175045, India

**Keywords:** Pain, Nociception, Ion channel, Natural product, Extract, Plant

## Abstract

Chronic pain affects approximately one-fifth of people worldwide and reduces quality of life and in some cases, working ability. Ion channels expressed along nociceptive pathways affect neuronal excitability and as a result modulate pain experience. Several ion channels have been identified and investigated as potential targets for new medicines for the treatment of a variety of human diseases, including chronic pain. Voltage-gated channels Na^+^ and Ca^2+^ channels, K^+^ channels, transient receptor potential channels (TRP), purinergic (P2X) channels and acid-sensing ion channels (ASICs) are some examples of ion channels exhibiting altered function or expression in different chronic pain states. Pharmacological approaches are being developed to mitigate dysregulation of these channels as potential treatment options. Since natural compounds of plant origin exert promising biological and pharmacological properties and are believed to possess less adverse effects compared to synthetic drugs, they have been widely studied as treatments for chronic pain for their ability to alter the functional activity of ion channels. A literature review was conducted using Medline, Google Scholar and PubMed, resulted in listing 79 natural compounds/extracts that are reported to interact with ion channels as part of their analgesic mechanism of action. Most *in vitro* studies utilized electrophysiological techniques to study the effect of natural compounds on ion channels using primary cultures of dorsal root ganglia (DRG) neurons. *In vivo* studies concentrated on different pain models and were conducted mainly in mice and rats. Proceeding into clinical trials will require further study to develop new, potent and specific ion channel modulators of plant origin.

## Highlights

• Voltage-gated channels (Na^+^, K^+^, Ca^2+^ channels), transient receptor potential channels (TRP), purinergic (P2X) channels and acid-sensing ion channels (ASICs) are heavily involved in both acute and chronic pain.
• Natural products of plant origin have been identified as novel treatments for pain and many act by targeting these ion channels.
• Here we review 79 natural compounds/extracts that are reported to interact with ion channels as part of their analgesic mechanism of action.
• Further study is required to develop new plant-based compounds towards clinical trials to develop novel analgesics.

## Introduction

Chronic pain affects approximately one-fifth of people worldwide and reduces quality of life and in some cases working ability. It is a global public health problem and a leading cause of disability all over the world (59 Global Prescription Medication Statistics: Strong Growth and CNS Well Represented, 2015, Gaskin and Richard, 2012, Stevens and Stephens, 2018). Nonsteroidal anti-inflammatory drugs (NSAIDs), tricyclic antidepressants (TCAs), anticonvulsants, muscle relaxants, and opioids are often prescribed as pharmacologic treatments of pain; however, their adverse effects, especially after long-term use, including gastrointestinal bleeding, the renal function destruction, and clinical tolerance and dependence strongly limit their application (Allegaert et al., 2010, Benyamin et al., 2008, Dowell et al., 2016, Duca et al., 2022, Hassett et al., 2014, Lapeyre-Mestre et al., 2013, Trang et al., 2015, Woolf and Hashmi, 2004). Considering the above, new therapeutic agents with increased efficacy, less side effects, and lower costs and leading to an improved quality of life should become one of the primary objectives in modern medical research (Daniyal and Wang, 2021, Johansen et al., 2012, Tasneem et al., 2019, van Hecke et al., 2014).

Plants contain a vast natural supply of compounds that may be a source of novel drugs. The medicinal use of plants as analgesic drugs in alternative medicine is far older than the current sciences of medicine in developed countries. Since the 1880′s, the most popular active ingredients to treat pain have been morphine (which comes from opium and poppies), salicylic acid (which comes from the bark of the white willow tree), and THC (which comes from cannabis) (Brune, 2002, Goyal, 2014, Goyal et al., 2013, Jones, 2011, Rivera et al., 2005, Zeb and Lee, 2021). In recent years, the exploration for new therapeutic agents capable of inhibiting, decreasing, or relieving pain with few or no adverse effects from the enormous arrays of medicinal plant resources is growing. Therefore, the present review summarizes the evidences of analgesic abilities of natural compounds/extracts from plant origin with activity towards the ion channels.

**Fig. 1:**
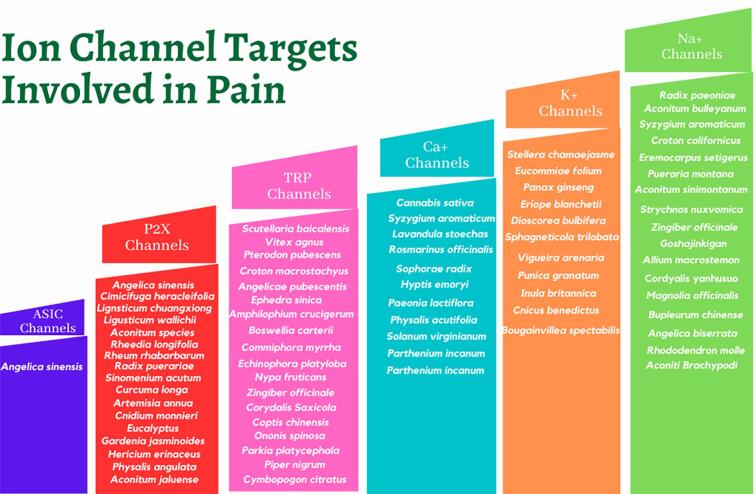
Natural products targeting Ion channels involved in pain.

Ion channels located at the nociceptor sensory peripheral terminal, facilitate the initiation of the signaling cascade in response to any noxious stimuli, affecting neuron excitability by altered action potential generation and propagation, axonal conduction and neurotransmitter release and further neuronal processing produces the experience of pain (Skerratt and West, 2015, Zhang et al., 2022). The role of voltage gated Na^+^ and Ca^2+^ channels, K^+^ channels, transient receptor potential channels (TRP), purinergic channels (P2X) and acid-sensing ion channels (ASICs) have been identified and investigated as potential targets for new medicines for the treatment of a variety of human diseases as well as acute and chronic pain (Bear et al., 2009, Bennett et al., 2019, Bernier et al., 2018, Birch et al., 2004, Cardoso and Lewis, 2018, Du et al., 2018, Du and Gamper, 2013, Lee and Chen, 2018, Markman and Dworkin, 2006, Moore et al., 2018, Ocaña et al., 2004, Takeda et al., 2011, Tsantoulas and McMahon, 2014, Zamponi et al., 2009) [Fig. 1]. Voltage-gated Na^+^ and Ca^2+^ ion channels are associated with setting neuronal excitability. VGSCs play a major role in action potential generation while the VGCCs control release of neurotransmitters. K^+^ channels are crucial in shaping action potentials and controlling the membrane potential, in excitable tissues including nociceptive sensory neurons, as a result of nerve or tissue injury (Bear et al., 2009, Bennett et al., 2019, Birch et al., 2004, Cardoso and Lewis, 2018, Du et al., 2018, Du and Gamper, 2013, Markman and Dworkin, 2006, Takeda et al., 2011, Zamponi et al., 2009). TRP channels are thermosensitive ion channels and have activation thresholds within the noxious range of temperatures (below 15 °C or above 43 °C) indicating possible involvement in thermal nociception, whereas the P2X channels are activated by extracellular ATP released from damaged or inflamed cells to initiate nociceptive signals (Bernier et al., 2018, Moore et al., 2018). Tissue acidosis is associated with inflammation and decreased extracellular pH (below pH = 6) opens ASIC channels resulting in activation of nociceptors (Lee and Chen, 2018).

Although a large number of small molecules have been reported to alter the functional activity of these ion channels, the effect and mechanism of action of natural products on these channels are still a matter of investigation. Here we focus on the effects of natural products on different ion channels involved in pain processing. We discuss sources, active constituents and chemical structures (where known) of natural products and their reported ion channel targets.

## Ion channel and drug targeting

Voltage gated Na^+^ and Ca^2+^ channels, K^+^ channels, transient receptor potential channels (TRP), purinergic channels (P2X) and acid-sensing ion channels (ASICs) are some of the ion channels classically involved in the pathogenesis of pain (Bear et al., 2009, Bennett et al., 2019, Bernier et al., 2018, Birch et al., 2004, Cardoso and Lewis, 2018, Du et al., 2018, Du and Gamper, 2013, Lee and Chen, 2018, Markman and Dworkin, 2006, Moore et al., 2018, Takeda et al., 2011, Zamponi et al., 2009).

### Voltage-gated sodium channels (VGSCs)

are important determinants of sensory neuron excitability: they are essential for the initial transduction of sensory stimuli, the electrogenesis of the action potential, and neurotransmitter release from sensory neuron terminals. Their activation depolarizes the resting membrane potential to generate an action potential upstroke. Na+ channels consist of a pore-forming α-subunit, as well as associated β-subunits. The family of related α-subunits consists of 10 members, 9 of which (Nav1.1–1.9) are voltage gated and one further non-voltage-gated member, Nax, which is involved in salt sensing. Nav1.1, Nav1.6, Nav1.7, Nav1.8, and Nav1.9 are all expressed by sensory neurons. The biophysical characteristics of these channels, as well as their unique expression patterns within subtypes of sensory neurons, define their functional role in pain signaling. Changes in the expression of VGSCs, as well as posttranslational modifications, contribute to the sensitization of sensory neurons in chronic pain states (Alles et al., 2020, Bennett et al., 2019, Black et al., 2012, Cox et al., 2006, Dib-Hajj et al., 2009, He et al., 2010, Luiz and Wood, 2016, Wang et al., 2011). Furthermore, gene variants in Nav1.7 (Alles et al., 2020, Bennett et al., 2019, Black et al., 2012, Cox et al., 2006), Nav1.8 (Bennett et al., 2019, He et al., 2010), and Nav1.9 (Bennett et al., 2019) have now been linked to the common pain disorders.

#### Effect of natural products on Voltage-gated sodium channels (VGSCs) (See Table 1)

##### Radix paeoniae

The effect of whole extract of *Radix paeoniae* (RP) on sodium currents (I_Na_) was examined in freshly isolated rat hippocampal CA1 neurons using whole-cell patch-clamp. The results suggested that the whole extract of RP suppressed hippocampal CA1 I_Na._ This mechanism was driven by a shift in the inactivation curve towards hyperpolarization. The effect was decreased recovery time from inactivation which attenuated the number of activity-dependent activatable channels in a dose dependent manner. Thus, RP whole extract may be used to reduce neuronal hyperexcitability (Dong and Xu, 2002).

**Table 1:** Anti-nociceptive potential of natural products from plant origin by modulating the ion channels activity.

Plant Product/Chemical Nature	Structure	Source	Target	Study Method	Type of pain/Sex/Species/Genetic background	Reference
Crude Extract	N/A	*Radix paeoniae*	Suppress voltage dependent sodium current	Whole cell patch clamp 0.8 mg/ml	Male-female/Wistar rat/Wild type	(Dong and Xu, 2002)
Bulleyaconitine A/ Diterpenoid alkaloid		*Aconitum bulleyanum*	Inhibit Nav1.3 and Nav1.7	Pituitary GH3 cells tested with 10 μM. Sensory and motor block of rat sciatic nerve, tested with 0.375 to 0.75 mM Rat spared nerve injury model of neuropathic pain IC50: Nav1.3, Nav1.7, and Nav1.8 were 995.6 ± 139.1 nM, 125.7 ± 18.6 nM, and 151.2 ± 15.4 lM, respectively	Male/SD rats/Wild types Chronic pain/Male/SD rats/Wild type	(Wang et al., 2007, Xie et al., 2018a, Xie et al., 2018b)
Eugenol/Phenolic essential oil		*Syzygium aromaticum*	Suppress voltage dependent sodium current	Dental primary afferent neurons. IC50 = 0.6 mM Chronic constriction injury (CCI) Dose: 10 & 50 μg, i.t.	SD rats/Wild type Chronic pain /SD rats/ Wild type	(Lionnet et al., 2010, Park et al., 2006)
(-)-Hardwickiic acid ((-)-HDA)/ Diterpenoid		*Croton californicus*	Inhibit Nav1.7	Rat DRGs tested with 20 μM. HEK cells tested with 20 μM. Alleviates HIV- and chemotherapy- induced neuropathy Dose: 2 μg/5 μl.	Chronic pain /Male-female/SD rats/Wild type	(Cai et al., 2018)
Hautriwaic acid (HTA)/ Diterpenoid		*Eremocarpus setigerus*	Suppress voltage dependent sodium current	Rat DRGs tested with 20 μM. Alleviates HIV- and Chemotherapy- induced neuropathy Dose: 2 μg/5 μl.	Chronic pain /Male -female/SD rats/Wild type	(Cai et al., 2018)
Puerarin/ Isoflavonoid		*Pueraria montana*	Inhibit Nav1.8	Rat DRG neurons IC50 = 481.5 μM. Paclitaxel-induced neuropathic pain at 8 mg/kg Dose: 0.1,1.0,10 uM, i.t.	Chronic pain /Male/SD rats/Wild type	(Zhang et al., 2018a)
Lappaconitine/ Diterpene alkaloid		*Aconitum sinimontanum*	Inhibit Nav1.7	HEK293 cells (Acute 30, 60, 100 μM). IC50 = 27.67 μM.	HEK293 cells	(Liao et al., 2019)
Brucine/ Second abundant alkaloid		*Strychnos nuxvomica*	Suppress voltage dependent sodium current	Chronic constriction injury (CCI) mouse model Dose: 10, 30 mg/kg	Chronic pain /Male/C57Bl/6 mice/Wild type	(Yu et al., 2019)
Gingerol and Shogaol/Beta-hydroxy ketone Phenol		*Zingiber officinale*	Inhibit Nav1.8	Rat oral ulcerative mucositis model HEK293 cells Human CHO cells 300 μM and 150 μM. Spinal nerve ligation (SNL) Dose: 100–400 mg/kg, oral	HEK293 cells Human CHO cells Chronic pain /Male/SD rats/Wild type	(Hitomi et al., 2017, Shen et al., 2022)
Neoline/ Alkaloid		Goshajinkigan extract formulation	Inhibit Nav1.7	HEK293 cells (20 mg/ml)Streptozotocin (STZ) -induced diabetic neuropathy Dose: 7.5 mg/kg	Chronic pain /Male/ICR mice	(Nakatani et al., 2020)
Crude extract	N/A	*Allium macrostemon*	Inhibit Nav1.7	Human embryonic kidney 293 T (HEK293T) 50 mg/L. Formalin-induced, Acetic-acid-induced and Thermal pain. Dose: 50 and 100 mg/kg/i.p.	Acute pain/Male/C57Bl/6 mice/Wild type	(Yang et al., 2021b)
L-Tetrahydropalmatine and protopine/ Alkaloids		*Cordyalis yanhusuo*	Inhibit Nav1.7	CHO cells IC50 = 7.05 μM. Formalin-induced pain model. Dose: 10,20 and 40 mg/kg,i.p.	Acute pain/ mice/wild type	(Xu et al., 2021a)
Magnolol/ Hydroxylated biphenyl		*Magnolia officinalis*	Suppress voltage dependent sodium current	NG108-15 cells. IC50 = 15 and 30 μM DRG neurons, TTX-S IC50 = 9.4 μM. TTX-R IC50 = 7 μM	NG108-15 cells Male/ICR mice	(Gong et al., 2012, Qiu et al., 2021)
Saikosaponins A/ Pentacyclic triterpenoid		*Bupleurum chinense*	Inhibit Nav1.7	Nav1.7 CHO cells IC50 = 28.6 nM Analgesic activity in thermal and formalin-induced pain in mice. Dose: 2.5,5.0,10.0 mg/kg, i.p.	Acute pain/Male -Female/Kunming mice	(Xu et al., 2021b)
Imperatorin/ Pentacyclic triterpenoid		*Angelica biserrata*	Inhibit Nav1.7	Nav1.7 CHO cells Thermal and formalin induced nociception Dose: 3.8,7.5,15.0 mg/kg, i.g.	Acute pain/Male -Female/Kunming mice	(Xu et al., 2021b)
Rhodojaponin III/ Grayanane-type diterpenoid		*Rhododendron molle*	Inhibit Nav1.7 and Nav1.8	hNaV1.5-CHL, hNaV1.7HEK293 and hNaV1.8HEK293 cell lines Thermal and acetic acid induced nociception Dose: 0.01–0.2 mg/kg Chronic constriction injury (CCI) Dose: 0.075,0.15,0.3 mg/kg	Acute-chronic pain/Male -Female/Kunming mice Male -Female/SD rat/Wild type	(Yang et al., 2022)
Ethanolic Extract	N/A	*Aconiti Brachypodi*	Suppressed TTX-sensitive sodium current	DRG culture Whole cell patch clamp 10 µg/ml-8.0 mg/ml Thermal and acetic acid induced nociception Dose: 1.0–20.0 mg/kg, i.g.	Male -Female/Wistar rat/Wild type Acute pain/Female/Kunming mice	(Ren et al., 2012)
Neochamaejasmin A (NCA)/ biflavonoid		*Stellera* *chamaejasme*	Modulate Kv1.4 channels	Human Kv1.4 CHO cell lines Whole cell patch clamp IC_50_ of 7.55 µM	Human Kv1.4 CHO cell lines	(Ren et al., 2018)
Chlorogenic acid (CGA)/ flavonoid		*Eucommiae folium*	Modulate I_K,A_ and I_K,V_ channels	TG neurons culture Whole cell patch clamp Dose: 0.2 and 1 mmolL^-1^	Male/SD rats/Wild type	(Zhang et al., 2014)
Gintonin/ ginseng saponins		*Panax ginseng*	inhibited Kv1.2 channel	Xenopus oocytes IC_50_ 0.58 ± 0.4 ug/mL	Xenopus laevis frog oocytes	(Lee et al., 2013)
Oleanolic acid / Pentacyclic triterpene		*Eriope blanchetii*	Activation of ATP-gated K^+^ channels	Capsaicin induced nociception Dose: 10, 30 and 100 mg/kg, oral	Acute pain/Male/Swiss albino mice/Wild type	(Maia et al., 2006)
Methanolic extract	N/A	*Dioscorea bulbifera*	Activation of the NO-cyclic GMP-protein kinase G_ATP_-sensitive potassium channel	CFA, LPS, PGE_2_ and Capsaicin induced nociception Dose: 500 mg/kg, oralPartial ligation of sciatic nerve (PLSN) Dose: 500 mg/kg, oral	Acute-chronic pain/Male-female/Swiss mice/Wild type	(Nguelefack et al., 2010)
Kaurenoic acid/Diterpene		*Sphagneticola trilobata*	Activation of the NO-cyclic GMP-protein kinase G_ATP_-sensitive potassium channel	Phenyl-p-benzoquinone, acetic acid and formalin induced nociception Dose: 3–30 mg/kg, i.p.	Acute pain/Male/Swiss mice/Wild type	(Mizokami et al., 2012)
Pimaradienoic acid/Pimarane diterpene		*Vigueira arenaria*	Activation of the NO-cyclic GMP-protein kinase G_ATP_-sensitive potassium channel	Acetic acid, formalin and CFA induced nociception Dose: 1, 3 and 10 mg/kg, i.p.	Acute pain/Male/Swiss mice/Wild type	(Possebon et al., 2014)
Ellagic acid/ Polyphenolic secondary metabolite		*Punica granatum*	Activation of L-arginine/NO/cGMP/K_ATP_ channel pathway	Formalin induced nociception Dose: 30–300 μg/paw/i.pl.	Acute pain/Male/Wistar rats/Wild type	(Ghorbanzadeh et al., 2014)
Patuletin/ Trimethoxyflavone flavonoid		*Inula britannica*	Activation of L-arginine/NO/cGMP/K_ATP_ channel pathway	Acetic acid, glutamate and formalin induced nociception Dose: 30 mg/kg, i.p.	Acute pain/Male/Swiss albino mice/Wild type	(Zarei et al., 2018)
Methanolic extract	N/A	*Cnicus benedictus*	Modulation of L-arginine/ nitric oxide/cGMP/ATP-sensitive potassium channel pathway	Acetic acid and formalin induced nociception Dose: 150, 150 and 300 mg/kg, i.p.	Acute pain/Male/Wistar rats/Wild type	(Ahmadimoghaddam et al., 2020)
Methanolic extract	N/A	*Bougainvillea spectabilis*	Modulation of ATP-sensitive K^+^ channel	Acetic acid, glutamate and formalin induced nociception Dose: 50, 100 and 200 mg/kg, oral	Acute pain/Male/Swiss albino mice/Wild type	(Ferdous et al., 2020)
Essential oil	N/A	*Artemisia biennis*	Activation of L-arginine/NO/cGMP/K_ATP_ channel pathway	Acetic acid, glutamate and formalin induced nociception Dose: 30, 60 and 120 mg/kg, oral	Acute pain/Male/Swiss mice/Wild type	(Zarei et al., 2021)
Essential oil	N/A	*Bupleurum falcatum*	Activation of L-arginine/NO/cGMP/K_ATP_ channel pathway	Formalin induced nociceptionCervical spinal cord hemicontusion (CSC) Dose: 25, 50 and 100 mg/kg, oral	Acute-chronic pain/Male/Swiss mice/Wild type	(Ahmadimoghaddam et al., 2021)
Cannabidiol/Phytocannabinoid Camphene and alpha‑bisabolol/ Terpenes		*Cannabis sativa*	Inhibit Cav3.1 and Cav3.2	HEK293 cells & TG neurons culture HEK tsA-201 cells DRG neurons culture Whole-cell patch clamp Complete Freund’s Adjuvant (CFA) and formalin induced nociception Partial sciatic nerve ligation (PSNL)	Male/C57Bl/6 mice/Wild type Acute and Chronic pain /Male-female/C57Bl/6J miceMale Cacna1h mice (Cav3.2 null mice)	(Gadotti et al., 2021, Harding et al., 2023, Ross et al., 2008)
Eugenol/Phenolic essential oil		*Syzygium aromaticum*	Inhibit Cav3.1 and Cav3.2	HEK293 cells TG neurons culture Whole-cell patch clamp	SD rats/Wild type	(Seo et al., 2013)
Linalool/ Essential oil		*Lavandula stoechas and Rosmarinus officinalis*	Inhibit Cav3.2	HEK-293 T Whole-cell patch clamp Ca^2+^ imagingOlfactory receptor cells (ORCs) Newt retinal neurons *Cerebellar Purkinje cells*	Wister rats/Wild type	(El Alaoui et al., 2017, Narusuye et al., 2005)
Sophoraflavanone G/6-prenylnaringenin		*Sophorae radix*	Inhibit Cav3.1 and Cav3.2	Cav3.1HEK293 cells and Cav3.2HEK293 cells Partial sciatic nerve ligation Oxaliplatin induced neuropathy	Chronic pain /Male/Wistar rat ddy mice/C57Bl/6j miceCacna1h mice (Cav3.2 null mice)	(Sekiguchi et al., 2018)
Betulinic acid/ Pentacyclic triterpenoid		*Hyptis emoryi*	Suppress voltage dependent calcium current	DRG neurons culture Whole cell patch clamp Ca^2+^ imaging HEKtsA-201 cells Voltage clamp recording Chemotherapy induced peripheral neuropathy (CIPN) HIV associated peripheral neuropathy Partial sciatic nerve ligation (PSNL)	Chronic pain /Male-female/SD rats/Wild type	(Bellampalli et al., 2019)
Astilbin/Flavonoid		*Paeonia lactiflora*	Suppress voltage dependent calcium current	Acetic acid, hot plate and formalin induced nociception Dose: 10 and 30 mg/kg	Acute pain/Female/BALB/c mice	(Bi et al., 2019)
Physalin F/Secosteroid		*Physalis acutifolia*	Inhibit Cav2.2	DRG neurons culture Paclitaxel-induced peripheral neuropathy Spinal nerve ligation Dose: 2ug/5ul, i.t.	Chronic pain /Male/SD rats/Wild type	(Shan et al., 2019)
Ethanolic extract	N/A	*Solanum virginianum*	Inhibit Cav2.2	Chronic construction injury (CCI) Dose: 100 and 200 mg/kg, oral	Chronic pain /Male-female/Wistar rats/Wild type	(Verma et al., 2020)
Argentatin-C/Triterpene		*Parthenium incanum*	Inhibit Cav3.1 Cav3.2 Cav3.3 Nav1.7 Nav1.8 Nav1.9	HEK293 cells Rat DRG neurons culture Molecular docking Paw incision mouse model of postoperative pain	Acute pain/Female/SD rats/Wild type Male CD1 mice	(Duran et al., 2022)
Heantos-4	N/A	Mixture of organic herbs	Inhibit Cav3.1 and Cav3.3	hCav3.1Flp-In293 cells, hCav3.2Flp-In293 cells and hCav3.3Flp-In293 cells Whole-cell patch clamp Acute brain slice electrophysiology 1 mg/ml	Male-female/Wistar rats/Wild type	(Cain et al., 2016)
Baicalin/ Glycosyloxyflavone		*Scutellaria baicalensis*	Modulation of TRPV1 channels	DRG neuron culture Ca^2+^ imaging Chronic constriction injury (CCI) Dose: 15 & 30 µg/kg, i.p.	New-born SD rats Chronic pain/Male/SD rats/Wild type	(Sui et al., 2010, Wang et al., 2020)
Vitexin/Flavonoid		*Vitex agnus*	Modulation of TRPV1 channels	Acetic acid, formalin, Complete Freund’s Adjuvant (CFA), c*apsaicin and thermal induced nociception* Dose: 1.0,3.0,10.0 mg/kg/i.p.	Acute pain/Male/Swiss mice/Wild type	(Borghi et al., 2013)
Ethanolic extract	N/A	*Pterodon pubescens*	Modulation of TRPV1 and TRPA1 channels	Partial sciatic nerve ligation Dose: 30.0, 100 and 300 mg/kg, i.g. Capsaicin 5 µl, i.t. Cinnamaldehyde 5 µl, i.t.	Acute and chronic pain /Female/Swiss mice/Wild type	(Nucci-Martins et al., 2015)
Methanol/methylene chloride extract	N/A	*Croton macrostachyus*	Modulation of TRPV1 channels	Complete Freund adjuvant (CFA), PGE_2_ and Capsaicin induced nociception Partial sciatic nerve ligation (PSNL) Dose: 250 and 500 mg/kg, oral	Acute and chronic pain/Male-female/Swiss mice/Wild type	(Nguelefack et al., 2015)
Coumarins/Aromatic organic compound		*Angelicae pubescentis*	Modulation of TRPV1 channels	Spared nerve injury (SNI) Dose: 5,10 and 20 mg/kg, i.g.	Chronic pain/Male/SD rats/Wild type	(Li et al., 2017)
Crude extract	N/A	*Ephedra sinica*	Modulation of TRPV1 channels	Flp-In293 cells, mTRPV1/Flp-In293 cells Capsaicin induced nociception, 0.03–3.1 ug/paw/i.d. Dose: 0.3–10 mg/paw, intra-planter 3 mg/paw,i.d. Dose: 87.5–700 mg/kg, oral	Acute pain/Male/ddY mice	(Nakamori et al., 2017)
Crude extract and dichloromethane fraction	N/A	*Amphilophium crucigerum*	Modulation of TRPV1 channels	Hot water tail-flick, Capsaicin and Complete Freund adjuvant (CFA) induced nociception Partial sciatic nerve ligation (PSNL) Dose: Crd 30, 100 and 300 mg/kg, i.g. Dcm 3,10 and 30 mg/kg, i.g.	Acute and chronic pain/Male/ albino swiss mice	(De Prá et al., 2017)
Water extract of frankincense and myrrh	N/A	*Boswellia carterii* and *Commiphora myrrha*	Modulation of TRPV1 channels	DRG neurons culture Ca^2+^ imaging Hot water tail-flick and Capsaicin induced nociception Chronic constriction injury (CCI) Dose: 1.5 and 7.5 mg/kg, i.g.	Acute and chronic pain/Male/ C57Bl/6 mice/Wild type	(Hu et al., 2017)
Polyacetylene fraction	N/A	*Echinophora platyloba*	Modulation of TRPA1 channels	HEK293 cells Thermo-TRPs receptor assays	HEK293 cells	(Chianese et al., 2018)
Ethanolic Extract	N/A	*Nypa fruticans*	Modulation of TRPV1 channels	Sciatic nerve crush injury Dose: 500 mg/kg, oral	Chronic pain/Male/SD rats/Wild type	(Kang and Hyun, 2020)
Ginger Extract and 6-shogaol/Phenols Zingiber officinale		*Zingiber officinale*	Modulation of TRPV1 channels	STZ induced diabetic peripheral neuropathy Dose: Ginger extract 100, 200 and 400 mg/kg, oral 6-shogaol 5, 10 and 15 mg/kg, oral	Chronic pain/Male/Balb/C mice	(Fajrin et al., 2020)
Crude extract	N/A	*Corydalis Saxicola*	Modulation of TRPV1 channels	Cisplatin induced neuropathic pain Dose: 30, 60 and 120 mg/kg, oral	Chronic pain/Male/SD rats/Wild type	(Kuai et al., 2020)
Berberine/Alkaloid		*Coptis chinensis*	Modulation of TRPV1 channels	Cisplatin induced peripheral neuropathy (CIPN) Dose: 60, 90 and 120 mg/kg, orally Partial sciatic nerve ligation (PSNL)	Chronic pain/Male/ C57Bl/6 mice (Wild type)/ TRPV1 Knockout mice	(Meng et al., 2021, Yang et al., 2020b)
Methanolic extract	N/A	*Ononis spinosa*	Modulation of TRPV1 channels	Capsaicin induced nociception, Dose: 40 µg/paw, ipl Dose: 100 µg/paw, ipl	Acute pain/Male/Wistar Rats/Wild type	(Jaffal et al., 2021)
Lectin/ Heterogeneous group of proteins	N/A	*Parkia platycephala*	Modulation of TRPV1 channels	Formalin induced-temporomandibular joint pain Infraorbital nerve transection- induced neuropathic pain Capsaicin 40.93 µM; 5.0 µl Lectin 0.025 mg/mL, 5.0 µl (Zebrafish), 0.25 mg/kg, ip (Rat)	Acute and chronic pain/Male/Wild Zebrafish Swiss mice/Wistar rats/Wild type	(de Oliveira Leite et al., 2022)
Viphyllin (standardized extract)	N/A	*Piper nigrum*	Modulation of TRPV1 channels	Acetic acid-induced writhing test, Formalin-induced paw licking test, hot plate test, Tail flick test capsazepine 0.1 mg/kg, i.p. Viphyllin 10–50 mg/kg, ip	Acute pain/Male/Balb/C mice/Wild type	(Venkatakrishna et al., 2022)
Citral/Terpene		*Cymbopogon citratus*	Modulation of TRPV1, TRPM3 and TRPM8 channels	Formalin, Cinnamaldehyd, Menthol and Capsaicin induced orofacial nociception Dose: 0.1, 0.3, 1.0, 3.0, 10.0, 30.0, 100 and 300 mg/Kg, oral Formalin temporomandibular joint (TMJ) nociception, Mustard oil-induced craniofacial nociception and Infraorbital nerve transection- induced neuropathic pain (IONX) Dose: 0.1 mg/kg, oral	Acute and chronic pain/Swiss mice/Wistar rats/Wild type	(Alves Rodrigues Santos et al., 2022)
Sodium ferulate (SF)/Sodium salt of ferulic acid		*Angelica sinensis*, *Cimicifuga heracleifolia*, *Lignsticum chuangxiong*	Modulation of P2X3 receptor channels	Rat DRG neurons culture Whole cell patch clamp Chronic constriction injury (CCI) Dose: 50 and 100 mg/kg, i.p.	Chronic pain/Male/SD rats/Wild type	(Zhang et al., 2010, Zhang et al., 2008)
Tetramethylpyrazine/Alkaloid		*Ligusticum wallichii*	Modulation of P2X3 receptor channels	Controlled cortical impact (CCI) Dose: 4 mM/scHot-water immersion (Burn-injury pain) DRG neurons culture Whole cell patch clamp Dose: 100 mg/kg, ip	Chronic pain/Male/C57Bl/6 mice/SD rats/Wild type	(Gao et al., 2010, Gao et al., 2008, Wang et al., 2017)
Lappaconitine (LA)/ Aconitum alkaloid		Extracted from the plants of Aconitum species	Modulation of P2X3 receptor channels	DRG neurons culture Whole cell patch clamp Chronic constriction injury (CCI) Dose: 4 mg/kg, ip	Chronic pain/Male/SD rats/Wild type	(Ou et al., 2011)
Methanolic Extract and some fractions	N/A	*Rheedia longifolia*	Modulation of P2X7 receptor channels	Dye uptake assay Whole-cell patch clamp IC50 = 2 μg/mL	Acute pain/Mice/Wild type	(Santos et al., 2011)
Emodin/ Natural anthraquinone		*Rheum rhabarbarum*	Modulation of P2X2 and P2X3 receptor channels	Chronic constriction injury (CCI) Dose: 50 mg/kg, ip	Chronic pain/Male/SD rats/Wild type	(Gao et al., 2011)
Puerarin/Isoflavonoid		*Radix puerariae*	Modulation of P2X3 and P2X7 receptor channels	Chronic constriction injury (CCI) Dose: 100 mg/kg, ip Clinical studies	Chronic pain/Male/SD rats/Wild type	(Li et al., 2011, Xu et al., 2012, Zhang et al., 2013)
Sinomenine/Alkaloid		*Sinomenium acutum*	Modulation of P2X3 receptor channels	Carrageenan induced inflammation Photochemically induced sciatic nerve injury Photochemically induced spinal cord injury Dose: 20,40 and 80 mg/kg, ip HEK293 cells Whole cell patch clamp STZ induced diabetic neuropathy Dose: 40 mg/kg, ip	Acute-chronic pain/Male-female/SD rats/male C57Bl/6 mice//Wild type Chronic pain/Male/SD rats/Wild type	(Gao et al., 2013, Rao et al., 2017)
Curcumin/Beta-diketone		*Curcuma longa*	Modulation of P2X3 receptor channels	DRG neurons culture Whole cell patch clamp HIV-gp120-induced neuropathic pain Dose: 4 mg/ml,sl	Chronic pain/Male/SD rats/Wild type	(Zhao et al., 2017)
Artemisinin/ Sesquiterpene lactone		*Artemisia annua*	Modulation of P2X4 receptor channels	HEK293 cells Whole cell patch clamp Chronic constriction injury (CCI) Dose: 5 mg/kg, ip	Chronic pain/Male/SD rats/Wild type	(Ying et al., 2017)
Osthole/Derivative of coumarin		*Cnidium monnieri*	Modulation of P2X4 receptor channels	STZ induced diabetic neuropathy Dose: 20 mg/kg, ip	Chronic pain/Male/SD rats/Wild type	(Yuan et al., 2018)
1,8-cineole/ Monoterpene cyclic ether		Eucalyptus	Modulation of P2X3 receptor channels	Chronic constriction injury (CCI) Dose: 50 and 100 mg/kg, ig	Chronic pain/Male-female/SD rats/Wild type	(Zhang et al., 2018b)
Gardenoside/Natural reactive aglycone		*Gardenia jasminoides*	Modulation of P2X3 and P2X7 receptor channels	Chronic constriction injury (CCI) Dose: 300 umol/l, iv	Chronic pain/Male/SD rats/Wild type	(Yu et al., 2018a, Yu et al., 2018b)
Hesperidin/ Bioflavonoid		Citrus fruits (family Rutaceae)	Modulation of P2X3 receptor channels	HEK293 cells Whole cell patch clamp Chronic constriction injury (CCI) Dose: 50 mg/kg, ip	Chronic pain/Male/SD rats/Wild type	(Tao et al., 2019)
Crude extract	N/A	*Hericium erinaceus*	Modulation of P2X4 and P2X7 receptor channels	Human neuroblastoma SH-SY5Y cellsL5-spinal nerve ligation (SNL) Dose: 100 mg/kg, ig	Chronic pain/Male/C57BL/6 NARL mice	(Yang et al., 2020a)
Crude ethanolic extract	N/A	*Physalis angulata*	Modulation of P2X7 receptor channels	HEK-293 cells Mouse peritoneal macrophages culture Dye uptake assay Whole cell patch clamp ATP-induced paw edema Dose: 0.001–100 mg/kg, ip	Acute pain/Male/Swiss webster mice	(Arruda et al., 2021)
Gallic acid/Phenolic acid		Found in gallnuts, sumac, witch hazel, tea leaves, oak bark	Modulation of P2X7 receptor channels	Neonatal colorectal dilation (CRD) Dose: 20 mg/kg, ig HEK293 cells Whole cell patch clamp Chronic constriction injury (CCI) Dose: 100 mg/kg,ip	Chronic pain/Male/SD rats/Wild type	(Wen et al., 2022, Yang et al., 2021a)
Resveratrol/ polyphenol		Plant species, including aliments, such as grapes, peanuts, and wines	Modulation of P2X3 and P2X7 receptor channels	STZ-induced diabetic neuropathy Dose: 25,100 and 400 mg/kg, ig Partial sciatic nerve ligation (PSNL) HEK 293 cells Whole cell patch clamp HIV-gp120 induced neuropathy Dose: 30 mg/kg, ip DRG neurons culture Whole cell patch clamp Chronic constriction injury (CCI) Dose: 25 mg/kg, oral	Chronic pain/Male/SD rats/Wild type	(Cui et al., 2020, Guo et al., 2021, Wu et al., 2017, Xie et al., 2017)
Astragalin/Flavonoid		White stamen of flowers	Modulation of P2X4 receptor channels	HEK 293 cells Whole cell patch clamp Chronic constriction injury (CCI) Dose: 50 mg/kg, ig	Chronic pain/Male/SD rats/Wild type	(Wang et al., 2021)
Crude water extract	N/A	*Aconitum jaluense*	Modulation of P2X7 receptor channels	L5-Spinal nerve ligation (SNL) Dose: 10,30,100 and 300 μg/10 μl, it	Chronic pain/Male/SD rats/Wild type	(Yang et al., 2016)
Ethanolic extract	N/A	*Azadirachta indica*	Modulation of ASIC hannels	Glutamate, formalin, cinnamaldehyde, capsaicin, menthol and acidic saline induced nociception Dose: 0.5,1.0,2.5,5.0 and 10.0 mg/ml, 20 μl, ip	Acute pain/Male-female/Wild zebra fish	(Batista et al., 2018)

##### Aconitum bulleyanum

Bulleyaconitine A, a diterpenoid alkaloid isolated from *Aconitum bulleyanum* plants. Experimental studies have revealed that bulleyaconitine A at therapeutic doses potently inhibits peripheral and central sensitization driven by upregulation of protein kinase C and VGSCs in DRG neurons (M.-X. Xie et al., 2018a; M. X. Xie et al., 2018b). Bulleyaconitine A effect is enhanced dose dependently via blocking voltage dependent Nav1.3 and Nav1.7 channels in DRG neurons and therefore, inhibits the ectopic discharges (Wang et al., 2007). Together, bulleyaconitine A is able to suppress nociception by targeting the voltage dependent sodium channels.

##### Syzygium aromaticum

Eugenol, an essential oil from *Syzygium aromaticum* plant, inhibited action potentials and voltage dependent sodium current (I_Na_) in neurons contributes to its analgesic effect (Park et al., 2006). The studies demonstrated that eugenol may alleviate neuropathic pain, both allodynia and hyperalgesia in CCI rats, by acting on central sensitization. The most probable site of action is at the level of the dorsal horn of the spinal cord, a location implicated heavily in nociception (Lionnet et al., 2010).

##### Croton californicus

Hardwickiic acid a diterpenoid isolated from plant *Croton californicus* inhibited voltage dependent Nav1.7 channels in DRG neurons. Therefore, this compound may be used as an antagonist alleviating Nav1.7 activation in the presence of non-noxious stimuli (Cai et al., 2018).

##### Eremocarpus setigerus

Hautriwaic acid a diterpenoid isolated from plants *Eremocarpus setigerus* inhibited voltage dependent sodium channels in the DRG neurons may novel specific sodium channel antagonists for pain relief (Cai et al., 2018).

##### Pueraria montana

Puerarin is a major isoflavonoid isolated from the root of *Pueraria montana* (Kudzu root) which has been used traditionally for treatment of cardiovascular disorders and brain injury. Additionally, puerarin acts on the β1 subunit of Nav1.8 channels in DRG neurons to attenuate hyperexcitability in neuropathic rats. The suppression of voltage dependent sodium currents contributed to its anti-paclitaxel induced neuropathic pain effect (Zhang et al., 2018a).

##### Aconitum sinimontanum

Lappaconitine is a diterpene alkaloid isolated from *Aconitum sinimontanum* and widely employed in Chinese and Japanese medicine mainly for analgesic indications. Studies reported that lappaconitine irreversibly inhibited Nav1.7 channels in a voltage dependent manner. Nav1.7 was stably expressed in human embryonic kidney (HEK293) cells which supports its application as a potent analgesic (Liao et al., 2019).

##### Strychnos nuxvomica

*Strychnos nuxvomica* is grown extensively in South Asia. Brucine, the second abundant alkaloid constituent of *Strychnos nuxvomica*, alleviated thermal hypersensitivity and mechanical allodynia in CCI induced neuropathic pain. This kind of inhibition is due to brucine which inhibits voltage dependent sodium channels. The result is reduced excitability of DRG neurons through a reduction of action potential firing frequency (Yu et al., 2019).

##### Zingiber officinale

Root extract of *Zingiber officinale* rich in gingerols and shogaols, exhibit antagonistic effects on voltage dependent Nav1.8 channels in oral ulcerative mucositis (Hitomi et al., 2017) and SNL induced neuropathic pain (Shen et al., 2022). Therefore, both ingredients demonstrate inhibitory effects on the generation of action potentials in DRG neurons, which contributes to the analgesic effects of *Zingiber officinale* in neuropathic pain.

##### Goshajinkigan extract formulation

Goshajinkigan extract formulation (GJG), an aqueous extract of a combination of 10 herbal medicines, a traditional Japanese Kampo formula, has been demonstrated to have an ameliorative effect on diabetic and chemotherapy associated peripheral neuropathic pain. Kampo formulae are composed of two or more kinds of natural crude drugs, and the decoctions of their mixtures are generally administered. Neoline as the active ingredient of GJG demonstrated antinociceptive effect via the inhibition of Nav1.7 current in streptozotocin as well as oxaliplatin-induced neuropathic pain in mice (Nakatani et al., 2020).

##### Allium macrostemon

*Allium macrostemon* is an edible herb traditionally used for the treatment of thoracic pain, stenocardia, asthma and diarrhea. Crude extract of *Allium macrostemon* significantly reduced pain behaviors in rodent pain models. Moreover, *Allium macrostemon* significantly reduced the excitability of sensory neurons by inhibition of the voltage dependent Nav1.7 channel contributing to a reduction in the firing frequency of action potentials thus reducing peripheral neuronal excitability (Yang et al., 2021b).

##### Corydalis yanhusuo

L-Tetrahydropalmatine and protopine monomers derived from *Corydalis yanhusuo* were tested *in vivo* and *in vitro*, to determine their analgesic properties. The results demonstrated that both monomers showed strong analgesic activity and inhibited the peak currents, which promoted the activation and inactivation phases of Nav1.7 channels (Xu et al., 2021a).

##### Magnolia officinalis

Magnolol, a hydroxylated biphenyl compound isolated from the bark of *Magnolia officinalis*, showed inhibitory effect on voltage dependent sodium currents at sensory neurons in a concentration-dependent manner (Gong et al., 2012). In addition, Magnolol significantly postponed recovery of voltage dependent Na^+^ currents from inactivation and produced frequency dependent blocks of both subtypes of Na^+^ currents (Qiu et al., 2021). These results suggest that the inhibitory effects of magnolol on Na^+^ channels may contribute to its analgesic effect.

##### Bupleurum chinense

Saikosaponin A, monomer derived from *Bupleurum chinense* was tested *in vivo* and *in vitro*, to determine its analgesic properties. The results showed that Saikosaponin A in *Bupleurum chinense* inhibited the peak currents of Nav1.7 in a concentration-dependent manner, suggesting they may be potential inhibitors of Nav1.7, thus indicates analgesic potential. Further, the study demonstrated that Saikosaponin A made Nav1.7 more easily activated and made it more difficult for the cell to return to its resting membrane potential thus delaying the regulation process of Nav1.7 channel as a whole. However, this did not affect the inactivation state of the channel. *In vivo* study of Saikosaponin A showed analgesic potential in thermal pain test and formalin-induced pain test in mice (Xu et al., 2021b).

##### Angelica biserrata

Imperatorin, monomer derived from *Angelica biserrate*, was tested *in vivo* and *in vitro* to determine its analgesic properties. The results showed that Imperatorin in *Angelica biserrata* inhibited the peak currents of Nav1.7 in a concentration-dependent manner, suggesting analgesic potential. Further study demonstrated that Imperatorin modulated Nav1.7 activation and inactivation thresholds. *In vivo* studies showed analgesic potential of Imperatorin in thermal pain and formalin-induced pain tests in mice (Xu et al., 2021b).

##### Rhododendron mole

Rhodojaponin III, an active constituent of *Rhododendron mole*, significantly inhibited the latency of the nociceptive response in the hot plate, tail-immersion, acetic acid and formalin-induced pain tests. Furthermore, Rhodojaponin III improved hyperalgesia in CCI rats. Electrophysiological experiments demonstrated that Rhodojaponin III mildly blocks Nav1.7 and Nav1.8 sodium channels to different degrees in a significant dose-dependent manner to ameliorate nociceptive and peripheral neuralgia-associated pain. Hepatotoxicity and leukopenia are associated as chronic side effects with Rhodojaponin III, hence, further investigation of sub-acute toxicity is necessary (Yang et al., 2022).

##### Aconiti Brachypodi

Ethanolic extract of *Aconiti Brachypodi* produced dose-dependent analgesic effects on hot plate tests, acetic acid induced writhing test, and formalin test in mice. *In vitro* studies of *Aconiti Brachypodi* extract indicated that temperate concentrations of extract reduced TTX-sensitive peak sodium current amplitudes in a dose-dependent way in rat’s dorsal root ganglion neurons, suggesting the modulation of Ethanolic extract of *Aconiti Brachypodi* on the TTX-sensitive sodium currents involved in its intervention in the input of nociceptive information (Ren et al., 2012).

### K^+^ channels superfamily is a very

large group of ion channels. Voltage-gated potassium channels (VGKKs) are important physiological regulators of membrane potentials, action potential shape, and firing adaptation in excitable tissues including nociceptive sensory neurons. (Jain et al., 2003, Johnston et al., 2010, Lázaro-Ibáñez et al., 2001, MacKinnon, 2003, Ocaña et al., 2004, Ortiz et al., 2003, Tsantoulas and McMahon, 2014, Yamazumi et al., 2001). Recent studies in various pain models identified the voltage gated potassium channels and non-VGKK channels including calcium-activated (K_Ca_) or ATP-sensitive potassium (K_ATP_) channels as potential therapeutic targets for pain (Abd-Elsayed et al., 2019, Du and Gamper, 2013, Wickenden and McNaughton-Smith, 2009).

#### Effect of natural products on K^+^ channels (Table 1)

##### Stellera chamaejasme

Neochamaejasmin A (NCA), a biflavonoid, one of the main active ingredients in the plant roots of *Stellera chamaejasme*, inhibited Kv1.4 channels in whole cell patch clamp of transfected human Kv1.4 CHO cell lines with IC_50_ of 7.55 µM via direct binding to the pore domain. Three mutations, V549A, A553V and V560A, occurred inside the pore, were found to significantly alleviate the NCA blocking effects, suggesting that they are the important binding sites of NCA (Ren et al., 2018).

##### Eucommiae folium

Chlorogenic acid (CGA), a flavonoid, obtained from dried leaves of *Eucommiae folium*, decreased the peak current density of I_K,A_ channels in whole cell patch clamp of rat trigeminal ganglion (TG) neurons. It caused significant reduction in the activation and inactivation thresholds of I_K,A_ and I_K,V_ channels and exhibited a strong effect on the activation and inactivation velocities of I_K,A_ and I_K,V_ channels. These findings provided novel evidence, explaining the biological effects of CGA, especially regarding its anti-nociceptive action (Zhang et al., 2014).

##### Panax ginseng

Gintonin, devoid of ginseng saponins, prepared from leaves of *Panax ginseng*, inhibited Kv1.2 channel activity in two electrode voltage-clamp experiment, in reversible and concentration-dependent manners in Xenopus oocytes after injection of RNA encoding the human Kv1.2α subunit. Gintonin mediated regulation of Kv1.2 channel activity might explain one of the modulations of gintonin mediated neuronal activities in nervous system (Lee et al., 2013).

##### Eriope blanchetii

Oleanolic acid, pentacyclic triterpene, isolated from aerial part of *Eriope blanchetii* inhibited capsaicin evoked acute nociception in mice. The study suggested that its antinociceptive action is at least, in part, related to the activation of ATP-gated K^+^ channels (Maia et al., 2006).

##### Dioscorea bulbifera

The methanolic extract of *Dioscorea bulbifera* indicated significant antinociceptive effects in persistent pain induced by intraplantar injection of complete Freund’s adjuvant and on neuropathic pain induced by partial ligation of sciatic nerve. This study demonstrated the antinociceptive activities of *Dioscorea bulbifera* on both inflammatory and neuropathic pain and these effects may result, at least partially, from its ability to activate the NO–cGMP–ATP-sensitive potassium channels pathway (Nguelefack et al., 2010).

##### Sphagneticola trilobata

Kaurenoic acid is a diterpene isolated from *Sphagneticola trilobata*, which dose-dependently inhibited inflammatory nociception induced by acetic acid, phenyl-p-benzoquinone, complete Freund's adjuvant, or formalin. Results indicate that kaurenoic acid exhibits a consistent analgesic effect and that its mechanism involves the activation of the NO-cyclic GMP-protein kinase G_ATP_-sensitive potassium channel signaling pathway (Mizokami et al., 2012).

##### Vigueira arenaria

Pimaradienoic acid is a pimarane diterpene extracted at high concentration from *Vigueira arenaria*. Pimaradienoic acid dose dependently inhibited inflammatory nociception induced by carrageenan-induced paw edema, acetic acid, complete Freund's adjuvant, and formalin. The study data show that pimaradienoic acid exhibits an analgesic effect and that its mechanisms involve the activation of the NO-cyclic GMP-protein kinase G_ATP_-sensitive potassium channel signaling pathway (Possebon et al., 2014).

##### Punica granatum

Ellagic acid, a polyphenolic secondary metabolite isolated from *Punica granatum*, produced a dose related peripheral antinociception during late phases of the formalin test which is comparable with morphine. The purposed mechanism involves activation of the l-arginine/NO/cGMP/K_ATP_ channels pathway followed by hyperpolarization of primary afferent neurons (Ghorbanzadeh et al., 2014).

##### Inula britannica

Patuletin is a trimethoxyflavone flavonoid isolated from *Inula britannica*, demonstrated significant antinociception potential in pain assessment tests including acetic acid induced writhing, formalin and glutamate induced paw licking. The results indicated that patuletin exhibits an analgesic effect. Its mechanisms involve the activation of the NO-cyclic GMP-protein kinase G_ATP_-sensitive potassium channel signaling pathway (Zarei et al., 2018).

##### Cnicus benedictus

Methanolic extract of *Cnicus benedictus* exhibited an antinociceptive effect on acetic acid-induced writhing and tail-flick tests. The mechanism of *Cnicus benedictus* antinociception involved activation of the NO-cyclic GMP-protein kinase G_ATP_-sensitive potassium channel signaling pathway (Ahmadimoghaddam et al., 2020).

##### Bougainvillea spectabilis

The methanolic extract of *Bougainvillea spectabilis* indicated significant antinociception potential in pain assessment tests including acetic acid-induced writhing and formalin induced paw licking. The study data showed that *Bougainvillea spectabilis* exhibits potent peripheral antinociceptive effects and that its mechanisms involve the modulation of the NO-cyclic GMP-protein kinase G_ATP_-sensitive potassium channel signaling pathway (Ferdous et al., 2020).

##### Artemisia biennis

Essential oil derived from *Artemisia biennis* had significant anti-nociceptive activity in the acetic acid induced writhing, tail-flick and formalin and glutamate induced paw licking assays and mechanical allodynia induced by cervical spinal cord contusion. Study data output indicated activation of the L-arginine-NO-cGMP-K_ATP_ system as a result of the anti-nociceptive abilities of *Artemisia biennis* extract (Zarei et al., 2021).

##### Bupleurum falcatum

Essential oil obtained from *Bupleurum falcatum* showed significant anti nociceptive activity in the formalin induced paw licking and mechanical allodynia induced by cervical spinal cord contusion. The study data indicated that *Bupleurum falcatum* exhibits potent anti-nociceptive effect and that its mechanisms involve the modulation of the L-arginine-NO-cGMP-K_ATP_ system pathway (Ahmadimoghaddam et al., 2021).

### Voltage-gated Ca^2+^ channels (VGCCs)

are expressed in excitable cells including DRG neurons where they control the release of neurotransmitters and neuronal excitability. VGCCs are responsible for depolarization-induced influx of Ca^2+^, triggers consequent release of neurotransmitter from synaptic vesicles and increase excitability thus blocking or genetically deleting these channels in hyperexcitable nociceptive neurons may reduce net excitability. These channels are well established mediators of pain signals in primary afferent neurons. The Ca^2+^ channel is composed of pore forming α1 subunit and the auxiliary subunits; β, γ and α2δ. On the basis of α1 subunit, they fall into three categories as Cav1-3. (Bourinet et al., 2014, Dolphin, 2018a, Dolphin, 2018b, Field et al., 2006, Rettig et al., 1996, Sheng et al., 1996, Zamponi et al., 2015). N-type Ca^2+^ channels are localized to synaptic nerve terminals in laminae 1 and 2 of the dorsal horn where their opening results in the release of neurotransmitters while T-type VGCCs are likely localized to nerve endings where they regulate cellular excitability. Consequently, inhibition of N-type and T-type VGCCs has the propensity to mediate analgesia. (Alles and Smith, 2018, Montera et al., 2021, Todorovic and Jevtovic-Todorovic, 2013).

#### Effect of natural products on VGCCs (Table 1)

##### Cannabis sativa

Cannabidiol obtained from *Cannabis sativa*, at moderately hyperpolarized potentials, Cannabidiol inhibited peak Cav3.1 and Cav3.2 currents by about 45%, but were less potent on Cav3.3 channels. Cannabidiol produced a significant hyperpolarizing shift in the steady state inactivation potentials for each of the Cav3 channels, which accounts for inhibition of channel currents and analgesic potential (Harding et al., 2023, Ross et al., 2008). Camphene and alpha-bisabolol, terpenes, isolated from *Cannabis sativa*, significantly inhibited Cav3.2 channels expressed in HEK tsA-201 cells, as well as native T-type channels in mouse DRG neurons by inhibiting peak current in the low micromolar range, and mediated an additional small hyperpolarizing shift in half-maximal inactivation threshold. Both terpenes inhibited nocifensive responses in mice that had received an intraplantar injection of formalin, reduced thermal hyperalgesia in mice injected with CFA and also inhibited mechanical hypersensitivity induced by partial sciatic nerve ligation. These effects were absent in Cav3.2 null mice, indicated that these compounds mediated their analgesic properties by acting on Cav3.2 channels (Gadotti et al., 2021).

##### Syzygium aromaticum

Eugenol, an essential oil from *Syzygium aromaticum* plant inhibited Cav3.1, Cav3.2, and Cav3.3 channels in a concentration-dependent manner by negatively shifting the steady-state inactivation curves of the T-type channel isoforms. Eugenol showed little effect on the current kinetics of Cav3.1 and Cav3.2, but it accelerated the inactivation kinetics of Cav3.3 currents and reduction of channel availability enhanced eugenol inhibition sensitivity for Cav3.1 and Cav3.2, but not for Cav3.3. T-type currents recorded from rat TG neurons were inhibited by eugenol with a similar potency to Cav3.1 and Cav3.2 isoforms. These findings suggested that T-type Ca^2+^ channels are additional molecular targets for the pain relieving effects of eugenol (Seo et al., 2013).

##### *Lavandula stoechas* and *Rosmarinus officinalis*

Methanolic extract of *Lavandula stoechas* and *Rosmarinus officinalis* containing active constituent essential oils, linalool and rosmarinic acid, respectively, inhibited Cav3.2 channels in a concentration dependent manner by negative shift of the steady-state inactivation of Cav3.2 channels with no change in the activation properties. These results demonstrated that the Cav3.2 calcium channels are molecular target of the linalool and rosmarinic acid for their antinociception activity (El Alaoui et al., 2017, Narusuye et al., 2005).

##### Sophorae radix

Sophoraflavanone G isolated from *Sophorae radix*, reported to blocked Cav3.1 and Cav3.2 channels. In mice, sophoraflavanone G, abolished the mechanical allodynia following intraplantar administration of a hydrogen sulfide donor, strongly suppressed visceral pain and spinal ERK phosphorylation, and alleviated the neuropathic allodynia induced by partial sciatic nerve ligation or oxaliplatin. The data demonstrated that sophoraflavanone G blocks T-type calcium channels and alleviates neuropathic and visceral pain (Sekiguchi et al., 2018).

##### Hyptis emoryi

Betulinic acid (BA) found in *Hyptis emoryi* inhibited depolarization evoked calcium influx in DRG neurons predominantly through targeting low-voltage gated Cav3.2, Cav3.3 and high-voltage gated Cav2.2 calcium channels resulting in reduced spontaneous excitatory post synaptic currents and depolarization-evoked release of calcitonin gene-related peptide (CGRP) from lumbar spinal cord slices. Voltage clamp electrophysiology experiments revealed a reduction of Ca^2+^, but not Na^+^, currents in sensory neurons following BA exposure. BA showed reversed mechanical allodynia in chemotherapy and partial sciatic nerve ligation, induced peripheral neuropathy. All these results highlighted BA as a potential non-opioid therapy for management of chronic pain (Bellampalli et al., 2019).

##### Paeonia lactiflora

Astilbin (AB), mainly obtained from *Paeonia lactiflora*, showed analgesic activities via regulation of the Ca^2+^ channels. AB strongly reduced the expression levels of c-Fos and phosphorylated calmodulin-dependent protein kinase II (CaMKII) and c-Jun N-terminal kinase (JNK) in the mice brain. These effects correlated with changes of the Ca^2+^ channel and intracellular Ca^2+^ influx which indicated evidence that astilbin-mediated analgesia is related to Ca^2+^ channels (Bi et al., 2019).

##### Physalis acutifolia

The natural product physalin F, isolated from the *Physalis acutifolia*, demonstrated antinociceptive effects in models of inflammatory pain. Physalin F reported to blocks Cav2.2 (N-type) voltage-gated calcium channels in DRG neurons without any effect on Cav3 calcium channels, voltage-gated sodium and potassium channels. It inhibited the frequency of spontaneous excitatory postsynaptic currents (sEPSCs) in spinal cord slices and reversed tactile hypersensitivity in models of paclitaxel-induced peripheral neuropathy and spinal nerve ligation (Shan et al., 2019).

##### Solanum virginianum

Ethanolic extract of *Solanum virginianum* significantly debilitated hyperalgesia and allodynia in CCI rats. Further docking simulation studies of solasodine (active constituent in *Solanum virginianum* extract) revealed that solasodine properly positioned at Cav2.2 may inactivate calcium channels (Verma et al., 2020).

##### Parthenium incanum

Argentatin C obtained from *Parthenium incanum* blocked the activity of both voltage-gated sodium and T-type calcium channels in calcium imaging assays. Docking analysis predicted that argentatin C may bind to Nav1.7–1.9 and Cav3.1–3.3 channels. Furthermore, argentatin C reversed mechanical allodynia in a paw incision mouse model of postsurgical pain by reducing the Na^+^ and T-type Ca^2+^ currents as well as excitability in rat and macaque DRG neurons (Duran et al., 2022).

##### Heantos-4

Heantos-4, is a mixture of organic herbs developed in Vietnam, significantly inhibited Cav3.1 and Cav3.3 currents in whole-cell voltage clamp study on exogenously expressed T-type calcium channels. These findings indicated that Heantos-4 has selective effects on specific T-type calcium channel isoforms makes it possible candidate with antinociceptive potential (Cain et al., 2016).

### Transient receptor potential channels (TRP channels)

are wide collection of a gene family involved in pain and itch sensory function. This family is made up of ion channel proteins that function as non-selective cation-permeable channels, virtually all of which conduct Ca^2+^ (Nilius, 2007). They function as molecular sensors of multiple physical and chemical stimuli, including changes in pH, chemical irritants including pungent peppers, wasabi, mustard, and menthol, as well as thermal, mechanical, osmotic, and actinic (radiation) cues. The TRP superfamily is composed of 28 members divided into six subfamilies, classified as canonical (TRPC), vanilloid (TRPV), ankyrin (TRPA), melastatin (TRPM), polycystin (TRPP), and mucolipin (TRPML) (Vennekens et al., 2008). The involvement of the TRPV1 (Chen et al., 2009), TRPV2 (Cheng et al., 2007), TRPA1 (Cho et al., 2012) and TRPM8 (McKemy et al., 2002, Peier et al., 2002, Weyer and Lehto, 2017) channels in thermal nociception has been well documented. Temperatures below 15 °C or above 43 °C evoke thermal sensation accompanied by the sensation of pain and these channels exhibits distinct thermal activation thresholds (Caterina et al., 1997, Caterina and Julius, 2001, Moore et al., 2018, Vennekens et al., 2008). Moreover, it is clear that spinal as well brain synaptic plasticity is an important procedure for the pain transition from acute to chronic in which TRP channels play critical roles presynaptically and postsynaptically (Choi et al., 2016, Duitama et al., 2020, Kim et al., 2008).

#### Effect of natural products on transient receptor potential channels (TRP) (Table 1)

##### Scutellaria baicalensis

Intraperitoneal (i.p.) 16-day administration of baicalin, a glycosyloxyflavone isolated from *Scutellaria baicalensis*, significantly reduced the mechanical and thermal nociceptive responses induced by CCI surgery in rats in a dose-dependent manner. The mRNA expression levels of TRPV1 and TRPA1 were significantly increased in the DRG of CCI rats. Moreover, baicalin administration, reversed mRNA expression level of TRPV1 (Sui et al., 2010) and suppressed TRPV1 upregulation and phosphorylation of extracellular signal-regulated kinases (MAPK/ERK pathway) (Wang et al., 2020) in DRG neurons after peripheral nerve injury might account for the anti-nociceptive mechanism of baicalin.

##### Vitex agnus

Vitexin, a flavonoid extracted from *Vitex agnus*, dose-dependently inhibited pain-like behavior i.e. mechanical and thermal hyperalgesia induced by capsaicin (an agonist of TRPV1), demonstrated that Vitexin exhibits an analgesic effect by targeting TRPV1 channels (Borghi et al., 2013).

##### Pterodon pubescens

The antinociceptive effects of ethanolic extract of *Pterodon pubescens* were reported on mechanical and thermal hyperalgesia in neuropathic pain induced by partial sciatic nerve ligation in mice along with nociceptive response induced by TRPV1 and TRVA1 agonists (capsaicin and cinnamaldehyde, respectively). Results indicated that oral administration of ethanolic extract of *Pterodon pubescens*, attenuate neuropathic pain associated thermal and mechanical hyperalgesia, without inducing tolerance, along with significant inhibition of TRPV1 and TRPA1 channels activators. This study added evidence for the therapeutic potential of *Pterodon pubescens* in the management of neuropathic pain (Nucci-Martins et al., 2015).

##### Croton macrostachyus

Methanol/methylene chloride extract of *Croton macrostachyus* was tested on CFA-induced persistent thermal and mechanical pain, neuropathic pain induced by partial sciatic nerve ligation (PSNL), prostaglandin E_2_ induced acute mechanical hyperalgesia, as well as on nociception induced by capsaicin in mice. *Croton macrostachyus* induced long lasting and significant antihyperalgesic effects on CFA-inflammatory and PSNL-induced neuropathic pain, reduced the mechanical hyperalgesia induced by PGE_2_ and time dependently inhibited the capsaicin-induced nociception. The results indicated that *Croton macrostachyus* exerted anti-nociception potential through the modulation of TRPV1 channels (Nguelefack et al., 2015).

##### Angelicae pubescentis

Coumarins, isolated from dried roots of *Angelicae pubescentis*, significantly prevented neuropathic pain and attenuated the development of mechanical hypersensitivity induced by spared nerve injury in rat. Molecular profiling revealed that coumarins reduced the levels of proinflammatory cytokines tumor necrosis factor-α (TNF-α), interleukin-1β (IL-1β) and interleukin-6 (IL-6) and significantly attenuated the expression of TRPV1 and pERK in damaged DRG neurons (Li et al., 2017).

##### Ephedra sinica

Ephedra herb extract (EHE) of *Ephedra sinica* significantly increased the intracellular Ca^2+^ concentration in stable mouse TRPV1-expressing mTRPV1/Flp-In293 cells, which was inhibited by the TRPV1 antagonist BCTC, indicated that EHE activated TRPV1 channels. *In vivo* study demonstrated that EHE induced paw licking behavior in a dose-dependent manner which was also inhibited by TRPV1 antagonist BCTC. Administration of EHE before administration of capsaicin suppressed capsaicin-induced paw licking by regulating TRPV1 activity on sensory neurons, without affecting the physical performance of the mice (Nakamori et al., 2017).

##### Amphilophium crucigerum

Crude extract and dichloromethane fraction of *Amphilophium crucigerum* reported antinociceptive effect in the hot water tail-flick and capsaicin intraplantar tests. Furthermore, these preparations exhibited anti-nociceptive and anti-inflammatory effects in a chronic inflammatory pain model, CFA, and anti-nociceptive effects in neuropathic pain model in mice. Moreover, crude extract and dichloromethane fraction reduced capsaicin-induced Ca^2+^ influx and diminished the [^3^H]-resiniferatoxin specific binding to spinal cord membranes. Results supported the analgesic effect of *Amphilophium crucigerum* and suggested the presence of compounds that may act as TRPV1 antagonists (De Prá et al., 2017).

##### Water extract of frankincense and myrrh (WFM)

Frankincense and myrrh are widely used in clinics as a pair of herbs obtained from *Boswellia carterii* and *Commiphora myrrha*, respectively, for their synergistic effects that relieve pain. *In vivo* study showed that the nociceptive response in mouse by heat and capsaicin induced were relieved by WFM treatment. Calcium response to capsaicin was also decreased in DRG neurons of CCI mouse after WFM treatment Furthermore, thermal hypersensitivity and mechanical allodynia were also alleviated by WFM treatment in a CCI neuropathic pain model by reverting the TRPV1 expression at both the mRNA and protein levels in predominantly small-to-medium neurons. In conclusion, WFM alleviated CCI-induced mechanical allodynia and thermal hypersensitivity via modulating TRPV1 expression (Hu et al., 2017).

##### Echinophora platyloba

Polyacetylene fraction isolated from *Echinophora platyloba* were evaluated for their modulation of six thermo-TRP channels (TRPA1, TRPM8, TRPV2-4, TRPM8) and they revealed a selective activity on TRPA1, an ion channel involved in the mediation of neuropathic and inflammatory pain (Chianese et al., 2018).

##### Nypa fruticans

In a sciatic crush injury rat model, a significant level of antinociceptive effect was reported in the thermal hyperalgesia test in which ethanolic extract of *Nypa fruticans* was orally administered. Protein quantification of the sciatic nerve and L4–L6 spinal cord showed a decreased TRPV1 expression, the inflammatory expression factor, COX2, and proinflammatory factors in the test groups. These results indicated that *Nypa fruticans* affects anti-nociceptive and anti-inflammatory by controlling TRPV1 in sciatic neuropathic pain models (Kang and Hyun, 2020).

##### Zingiber officinale

Ethanolic extract of *Zingiber officinale* and its active constitute 6-shogaol, alleviated hyperalgesia and allodynia in the STZ induced diabetic peripheral neuropathy mice model. Both ginger extract (400 mg/kg) and 6-shogaol (15 mg/kg) significantly reduced TRPV1 and NMDAR2B expressions in the spinal cord with very limited effect on pancreatic islets, compared to the diabetic control group. TRPV1 functionally interacts with N-methyl-D-aspartate receptors (NMDAR) and contributes to the development of pain behavior. Research found that the expression of NMDAR subunit 2B (NMDAR2B) in the spinal cord's dorsal horn is higher in mice models of diabetic neuropathy (Fajrin et al., 2020).

##### Corydalis Saxicola

Crude extract of *Corydalis Saxicola* reported anti-nociception potential in cisplatin-induced mechanical, heat, and cold hyperalgesia. *Corydalis Saxicola* exerted its therapeutic effects by ameliorating neuronal damages, improving intraepidermal nerve fiber (IENF) loss, and inhibiting inflammation-induced p38 phosphorylation to block TRPV1 activation (Kuai et al., 2020).

##### Coptis chinensis

Berberine obtained from *Coptis chinensis* demonstrated to increase both mechanical and thermal pain thresholds in a dose-dependent manner in partial sciatic nerve ligation (PSNL) (Yang et al., 2020b) and cisplatin-induced (CIPN) peripheral neuropathy (Meng et al., 2021). The pain reducing potential of berberine exerted by reversed the mRNA and protein expression of TRPV1 in dorsal root ganglion neurons after peripheral nerve injury (Yang et al., 2020b). Moreover, berberine mediated the neuroinflammatory reaction induced by cisplatin by inhibiting the overexpression of TRPV1 and NF-κB and activating the JNK/p38 MAPK pathways in early injury, which inhibited the expression of p-JNK and mediated the expression of p38 MAPK/ERK in late injury *in vivo* at dorsal root ganglion neurons (Meng et al., 2021).

##### Ononis spinosa

Methanolic extract of *Ononis spinosa* alleviated capsaicin induced mechanical allodynia through the direct modulation of TRPV1 and the involvement of β2 adrenoreceptor signaling (Jaffal et al., 2021).

##### Parkia platycephala

Lectin isolated from *Parkia platycephala* reduced nociceptive behavior in adult zebrafish, and this is related to the activation of the TRPV1 channels since antinociception was effectively inhibited by capsazepine and by capsaicin-induced desensitization. Lectin reduced allodynic nociceptive behavior associated with formalin induced temporomandibular joint pain and infraorbital nerve transection induced neuropathic pain in rats. The results confirmed the potential pharmacological relevance of *Parkia platycephala* as an inhibitor of orofacial nociception in acute and chronic pain through the modulation of TRPV1 (de Oliveira Leite et al., 2022).

##### Piper nigrum

Viphyllin, a standardized extract of *Piper nigrum* seeds, was reported to significantly inhibit the acetic acid induced writhing and formalin induced paw licking. It increased the withdrawal latency in hot plate and tail flick test. Capsazepine abolished the analgesic effect of Viphyllin, clearly suggested the involvement of TRPV1 ion channel in Viphyllin mediated antinociceptive effect (Venkatakrishna et al., 2022).

##### Cymbopogon citratus

Citral, a naturally occurring terpene, extracted from *Cymbopogon citratus*, produced significant antinociception on acute nociceptive behaviors, and these effects were attenuated by TRPV1 antagonist capsazepine, TRPM3 antagonist mefenamic acid and by TRPM8 desensitization. The infraorbital nerve transection (IONX) animals developed facial mechanical hypersensitivity that was significantly reduced by citral. The docking experiments revealed that citral may interact with TRPV1 and TRPM8 channels. These results indicated the potential use of citral as an inhibitor of orofacial nociception in both acute and chronic pain states through TRPV1, TRPM3 and TRPM8 channels (Alves Rodrigues Santos et al., 2022).

### Purinergic receptor cation channels (P2X)

also known as the ATP-gated P2X receptor cation channel family that consists of seven receptor subtypes named P2X1-P2X7, is made up of cation-permeable ligand-gated ion channels that open in response to extracellular adenosine 5′-triphosphate binding (ATP). ATP released from damaged or inflamed cells activates the excitatory and calcium-permeable P2X receptor channels to initiate and maintain the nociceptive signals, therefore, their selective targeting represents a therapeutic opportunity for pain management. The P2X channels are reported to play crucial role in central nervous system pain transmission and persistent modulation upon and following the occurrence of neuropathic pain. Recent advances in the structural, functional and pharmacological characterization of rodent and human ATP-gated P2X receptor channels have shed brighter light on the role of P2X2, P2X3, P2X4 (Westlund et al., 2021) and P2X7 receptor channels in the pathogenesis of central pain including the mediation of fast transmission in the peripheral nervous system and modulation of neuronal activity in the central nervous system (Bernier et al., 2018, Gever et al., 2006, Khakh and Alan North, 2006, Kuan and Shyu, 2016, North, 2002).

#### Effect of natural products on purinergic receptor cation channels (P2X) (Table 1)

##### Sodium ferulate

Sodium ferulate (SF) is an active principle of *Angelica sinensis*, *Cimicifuga heracleifolia*, *Lignsticum chuangxiong* and expressed antioxidant and anti-inflammatory activities. SF indicated reduced thermal and mechanical hyperalgesia in CCI rat model by decreasing the pain transmitted by primary afferent neurons mediated by P2X3 receptor. In CCI rats treated with SF, the Mechanical withdrawal threshold, and thermal withdrawal latency were increased while the upregulated expression of P2X3 receptors in DRG neurons was reduced followed decreased the increment of P2X3 agonist-activated currents and P2X3 mRNA expression, compared to the normal saline group (Zhang et al., 2010, Zhang et al., 2008).

##### Ligusticum wallichii

Tetramethylpyrazine (TMP), an alkaloid, is an important compound in *Ligusticum wallichii*, reported to inhibit the primary afferent transmission of neuropathic pain induced by P2X3 receptor in CCI rats. TMP reduced the mechanical withdrawal threshold and thermal withdrawal latency by downregulation of the P2X3 receptor expression in L4/L5 DRG neurons and spinal cord (Wang et al., 2017). TMP reported to alleviates nociceptive transmission of burn-injury pain mediated by the P2X3 receptor (Gao et al., 2008, Gao et al., 2010).

##### Lappaconitine

Lappaconitine (LA) is an aconitum alkaloid extracted from the plants of genus *Aconitum*, showed increased pain thresholds, the down-regulated P2X3 receptor expression and the reduced P2X3 receptor agonists ATP- and α, β-meATP-induced inward currents (I_ATP_ and Iα, β-meATP) in the acutely dissociated rat DRG neurons of CCI rats. These results indicated that the analgesic effect of LA involves decreased expression and sensitization of the P2X3 receptors of the rat DRG neurons following CCI (Ou et al., 2011).

##### Rheedia longifolia

The *Rheedia longifolia* extract and some fractions showed an analgesic and anti-inflammatory activity by inhibitory effect on the P2X7 purinergic receptor in a dose-dependent manner. The ethyl acetate fraction exhibited the most potent inhibitory effects than others like methanol extract and the butanol fraction. Further investigation is needed to determine the pattern of inhibition and selectivity (Santos et al., 2011).

##### Rheum rhabarbarum

Emodin, an anthraquinone obtained from *Rheum rhabarbarum* extract, demonstrated anti-hyperalgesic potential associated with significant reduction of P2X2/3 expression of L4/L5 DRG neurons in CCI rats. The data of immunohistochemistry, in situ hybridization (ISH) and RT-PCR in P2X2 and P2X3 mRNA expression suggested that the antinociceptive mechanism of emodin is involved in the nucleic acid level (Gao et al., 2011).

##### Radix puerariae

Puerarin, an isoflavonoid, obtained from *Radix puerariae*, decreased the thermal and mechanical hyperalgesia by inhibiting the up-regulated expression of P2X3 receptors from DRG neurons of CCI rats (Xu et al., 2012). The inflammation and associated pain involved in dressing changes of burn patients were relieved by puerarin treatment and this effect were correlated with the decreased expression level of P2X3/7 receptors mRNA and protein in peripheral blood mononuclear cells (PBMCs) of burn patients (Li et al., 2011, Zhang et al., 2013).

##### Sinomenium acutum

Sinomenine, an alkaloid originally isolated from the root of the plant *Sinomenium acutum*, significantly inhibited P2X3 agonist ATP-activated currents in HEK293 cells transfected with the P2X3 receptor. Sinomenine was reported to relieve the hyperalgesia in rats by suppressed the up-regulated expression and activation of the P2X3 receptor followed by decreased the phosphorylation and activation of P38MAPK in Type-2 diabetes mellitus (T2DM) inflicted DRG (Rao et al., 2017). In conclusion, sinomenine demonstrated potential to effectively alleviate mechanical and cold allodynia in rats and mice after photochemically induced sciatic nerve and spinal cord injury (Gao et al., 2013).

##### Curcuma longa

Study showed that peripheral nerve exposure to HIV gp120 increased neuropathy associated mechanical and thermal hyperalgesia accompanied by upregulated expression of the P2X3 receptor in the DRG of the gp120-treated model rats. Nano curcumin (*Curcuma longa*) treatment decreased the upregulated expression of the P2X3 receptor in DRG of gp120-treated model rats, followed by suppressed phosphorylation of ERK1/2 thus reduced the sensitization of DRG primary afferents and relieved mechanical and thermal hyperalgesia in gp120-treated rats (Zhao et al., 2017).

##### Artemisia annua

Artemisinin, extracted from *Artemisia annua* leaves, is a type of sesquiterpene lactone, relieved pain behaviors in the CCI rats, inhibited the expression of P2X4 receptor in the DRG, and decreased the ATP-activated currents in HEK293 cells transfected with P2X4 plasmid. Dual-labeling immunofluorescence study showed that the artemisinin significantly decreased the co-expression of P2X4 receptor and glial fibrillary acidic protein (GFAP) in DRG neurons of CCI rats (Ying et al., 2017).

##### Cnidium monnieri

Osthole is a component extracted from *Cnidium monnieri* plant seeds and has anti-inflammatory and anti-oxidative properties. Osthole treatment data showed decreased the P2X4 receptor upregulation and SGC activation in DRG neurons, followed by the down-regulation of IL1β, TNF-α, BDNF and p-p38MAPK and the up-regulation of IL-10 in diabetic mellitus (DM) rats. Osthole treatment may act on the P2X4 receptor to alleviate the mechanical and thermal hyperalgesia in DM rats (Yuan et al., 2018).

##### Eucalyptus

1,8-cineole is a natural monoterpene cyclic ether present in eucalyptus and has been reported to exhibit anti-inflammatory and antioxidant effects. 1,8-cineole treatment indicated decreased the mechanical withdrawal threshold and thermal withdrawal latency by down-regulation of P2X3 receptor mRNA expression and P2X3 receptor protein expression in the L4-L5 DRG neurons of CCI rats. These results demonstrated that 1,8-cineole can alleviate pathological pain caused by P2X3 receptor stimulation (Zhang et al., 2018b).

##### Gardenia jasminoides

Gardenoside, also known as genipin, is a natural reactive aglycone isolated from the fruit of *Gardenia jasminoides*, significantly improved the sciatica by partially restored the decreased of mechanical withdrawal threshold and thermal withdrawal latency in CCI rats. Further, results indicated that the levels of iNOS, IL-1β, TNF-α, p-ERK/ERK and p-p38/p38, and expressions of P2X3 and P2X7 receptors in the L4-L5 DRG neurons were significantly decreased in the CCI rats after gardenoside treatment. It was also reported that gardenoside combined with ozone could alleviated chronic neuropathic pain. The effects of gardenoside and ozone may be mediated by the inhibition of P2X3 and P2X7 receptor expression in the rat DRG (Yu et al., 2018a, Yu et al., 2018b).

##### Hesperidin

Hesperidin is a bioflavonoid, found in citrus fruits (family Rutaceae) with cardioprotective, neuroprotective, antioxidative and anti-inflammatory activities. Hesperidin reported to relieved the abnormal mechanical and thermal hyperalgesia in CCI rats by suppressed the upregulated expression of P2X3 protein and mRNA in DRG neurons which was accompanied by activation of ERK1/2 (Tao et al., 2019).

##### Hericium erinaceus

Crude extract of *Hericium erinaceus* reported to suppressed, the increased level of IL-6, activation of astrocytes and microglia and upregulated expression of P2X4 and P2X7 receptors at DRG neurons in L5-spinal nerve ligation mice model, thus relieved the neuropathic pain (Yang et al., 2020a).

##### Physalis angulata

Crude ethanolic extract of *Physalis angulata* enriched with physalin B, D, F, and G forms, showed dose-dependent inhibition of P2X7 receptor function and ATP-induced paw edema was potently inhibited in mice (Arruda et al., 2021).

##### Gallic acid

The results showed that CCI rats treated with gallic acid, the mechanical withdrawal threshold and thermal withdrawal latency were increased, accompanied by inhibition of the upregulated expression of P2X7 and TNF-α at both mRNA and protein levels, and reduced NF-κB and phosphorylated-STAT3 in the dorsal root ganglia. Gallic acid significantly decreased the co-expression of P2X7 and glial fibrillary acidic protein (GFAP) in the DRG. In addition, gallic acid could suppress ATP activated current in human embryonic kidney 293 (HEK293) cells transfected with the plasmid expressing P2X7 (Wen et al., 2022, Yang et al., 2021a).

##### Resveratrol

Resveratrol (RES) is a natural polyphenol obtained by a wide variety of plant species, including aliments, such as grapes, peanuts, and wines. The results suggested that RES ameliorated neuropathic pain in a dose-dependent manner induced by partial sciatic nerve ligation (PSNL) and STZ (DNP) in rats, by suppressing P2X3 up-regulation and ERK phosphorylation in DRG neurons and spinal dorsal horn terminals (SDH) (Cui et al., 2020, Guo et al., 2021). RES was also reported to decrease the sensitization of the P2X7 receptors in the satellite glial cells of DRG neurons after CCI and HIV envelope glycoprotein 120 (gp120) treated rats and increase the threshold of thermal and mechanical hypersensitivity in rats with chronic neuropathic pain (Wu et al., 2017, Xie et al., 2017).

##### Astragalin

Astragalin (AST), is a flavonoid extracted from the white stamen of some flowers, demonstrated partly abrogated the upregulation of P2X4, inhibited SGC activation, and alleviated pain behavior in CCI rats. It also suppressed ATP-activated currents in HEK293 cells overexpressing P2X4 (Wang et al., 2021).

##### Aconitum jaluense

Crude water extract of *Aconitum jaluense* showed anti-allodynic effects in neuropathic pain by suppression of P2X7 receptor expression as well as reduced microglial activation in the spinal cord of SNL rats (Yang et al., 2016).

### Acid-sensing ion channels (ASICs)

are voltage-independent depolarizing sodium channels, expressed in somatosensory neurons, belonging to the degenerin/ENaC superfamily and activated by extracellular protons. Six isoforms have been identified encoded by four different genes: ASIC1a, ASIC1b, ASIC2a, ASIC2b, ASIC3 and ASIC4. During inflammation, tissue damage and ischemia, the extracellular pH values decreases, which activates nociceptors by activating a particular pH-specific ASIC. All ASICs except ASIC4 are expressed in DRG neurons. ASIC subunits are differentially expressed in different DRG neuronal subtypes after nerve injury suggesting a role in different sensory modalities (Deval et al., 2010, Lee and Chen, 2018, Papalampropoulou-Tsiridou et al., 2020).

#### Effect of natural products on acid-sensing ion channels (ASICs) (Table 1)

##### Azadiractha indica

Ethanolic extract of *Azadiractha indica*, significantly inhibited the acute nociception induced by acidic saline (0.1%) in an adult zebra fish experimental model. There was no difference between these groups treated with extract or morphine or naïve ones. The antinociceptive effect of extract was abolished by amiloride suggested that the antinociceptive effect of this extract on acute pain seems to be modulated by the acid-sensing ion channels (ASIC channels) (Batista et al., 2018).

## Conclusion

Traditional medicine is used by different cultural groups all over the world and remedies have been passed down from generation to generation to maintain health. Ion channels like voltage-gated channels (Na^+^, Ca^2+^ channels), K^+^ channels, transient receptor potential channels (TRP), purinergic (P2X) channels and acid-sensing ion channels (ASICs) are critical for establishing acute and chronic pain and modulating the function of these channels can significantly alleviate pain. In recent years, natural medicinal agents of plant origin possess ion channel-modulating potential and have been recognized as a valuable source of new therapeutics for pain management. This review summarizes 79 natural products (53 isolated compounds and 26 crude extracts or formula) based on 97 research articles that show potent analgesic potential with activity at ion channels. Some of these natural products have undergone clinical trials, while others warrant further investigation for their mechanisms on pain signaling pathways. Most of the compounds/extracts identified did not present any toxicity or known side effects and were at least as efficient as currently used synthetic drugs. Out of 97 research articles, 30 articles used both male and female rodents while 52 used males, 4 used female rodents, and the remaining 11 articles are based on cell lines. Few studies mentioned in this review have used more than one species of rodents for different experiments to assess the anti-nociceptive activity of natural products. A total 55 studies used rats (wild type) of which 43 used Sprague Dawley rats and 12 are Wistar rats. Forty studies used mice of which 11 used C57Bl/6 mice, 2 used ICR mice, 4 used Kunning mice, 14 used Swiss albino mice, 2 used ddy mice, 3 used Balb/c mice, 1 used CD1 mice and 3 used knockout-mice. Three studies used non-mammalian models and five studies used human ion channel-expressing CHO cells. This comprehensive review, addressed both acute and chronic pain studies. Twenty-two studies are on acute pain models, 34 studies are on various chronic pain models (HIV-related, chemotherapy-induced and diabetic neuropathies, nerve injury and spinal cord injury), and 12 studies address both acute and chronic pain models. Future studies should focus on investigating mechanisms of action, dose ranges, clinical efficacy, safety of the extracts, sex as a biological variable, and active constituents to find more specific and safer molecules to target ion channels. In addition, more studies should be carried out on human neuronal cells to address translational potential (Renthal et al., 2021). However, the findings of this review are promising regarding the development of new potential therapeutic agents from natural products for treating acute and chronic pain.

## Declaration of Competing Interest

The authors declare that they have no known competing financial interests or personal relationships that could have appeared to influence the work reported in this paper.
